# Perspectives in the use of tannins as alternative to antimicrobial growth promoter factors in poultry

**DOI:** 10.3389/fmicb.2014.00118

**Published:** 2014-03-27

**Authors:** Leandro M. Redondo, Pablo A. Chacana, Johana E. Dominguez, Mariano E. Fernandez Miyakawa

**Affiliations:** ^1^Instituto de Patobiología, Centro de Investigación en Ciencias Veterinarias y Agronómicas, Instituto Nacional de Tecnología AgropecuariaBuenos Aires, Argentina; ^2^Consejo Nacional de Investigaciones Científicas y TécnicasBuenos Aires, Argentina

**Keywords:** tannins, antibiotics, poultry, growth promoting factors, necrotic enteritis, plant extracts, animal health

## Abstract

Antibiotics have been included in the formulation of feed for livestock production for more than 40 years as a strategy to improve feed conversion rates and to reduce costs. The use of antimicrobials as growth-promoting factors (AGP) in sub-therapeutic doses for long periods is particularly favorable for the selection of antimicrobial resistant microorganisms. In the last years, global concern about development of antimicrobial resistance and transference of resistance genes from animal to human strains has been rising. Removal of AGP from animal diets involves tremendous pressure on the livestock and poultry farmers, one of the main consequences being a substantial increase in the incidence of infectious diseases with the associated increase in the use of antibiotics for therapy, and concomitantly, economic cost. Therefore, alternatives to AGP are urgently needed. The challenge is to implement new alternatives without affecting the production performances of livestock and avoiding the increase of antimicrobial resistant microorganisms. Plant extracts and purified derived substances are showing promising results for animal nutrition, either from their efficacy as well as from an economical point of view. Tannins are plant derived compounds that are being successfully used as additives in poultry feed to control diseases and to improve animal performance. Successful use of any of these extracts as feed additives must ensure a product of consistent quality in enough quantity to fulfill the actual requirements of the poultry industry. Chestnut (hydrolysable) and Quebracho (condensed) tannins are probably the most readily available commercial products that are covering those needs. The present report intends to analyze the available data supporting their use.

## INTRODUCTION

Antimicrobial compounds were initially added to feed at therapeutic doses for treatment and prevention of infectious diseases but soon the growth promoting effect of antibiotics was observed. Therefore, since the beginning of the 1950s antibiotics have been added to feeds to improve feed utilization and growth of farm animals, reducing the cost of production (Moore and Evenson, 1946; Jukes et al., 1950). The use of antimicrobials as growth-promoting factors (AGP) should be distinguished from therapeutic and prophylactic use of antibiotics that are administrated at higher doses and for short periods of time.

The mode of action of AGPs is not yet fully understood. Different potential mechanisms have been proposed to explain AGP-mediated growth enhancement (Gaskins et al., 2002; Dibner and Richards, 2005; Page, 2006). The most accepted mechanism would be through modulation of the gut microbiota, which plays a critical role in maintaining the host health (Tuohy et al., 2005). Microbiota composition influences the intestinal environment and the development and responses of the host immune system against pathogenic and non-pathogenic antigens (Cebra, 1999; Kelly and Conway, 2005).

The poultry industry has massively adopted the use of AGPs, but comparatively, little research has been conducted in order to systematically evaluate the potential effects that antibiotics may have on the dynamics of the overall gut microbiota of chicken. Thus, studies are indispensable to elucidate the impact on bacterial community, including selection and distribution of antibiotic resistance genes among commensal bacteria in chickens fed with AGP (Diarra et al., 2007; da Costa et al., 2013).

The use of AGP in livestock and their role in selecting antibiotic resistant bacteria have been extensively reviewed (Butaye et al., 2003; Wegener, 2003; Kazimierczak et al., 2006; Landers et al., 2012). It is important to remark that AGP are used in sub-therapeutic doses for long periods, a situation that is particularly favorable to select antimicrobial-resistant microorganisms. During the last several years, global concern about development of antimicrobial resistance and transference of resistance genes from animal to human strains is rising (Salyers et al., 2004; Mathur and Singh, 2005; Devirgiliis et al., 2013). The potential risk of resistance generation and transmission led to the ban of the use of antibiotics as growth promoters in the European Union since year 2006. Although the relative contribution of foodborne transmission to antimicrobial resistance in humans remains unknown, it does exist and is likely to be more substantial than currently appreciated (Collignon and Angulo, 2006). Some studies suggest that the majority of antibiotic-resistant *Escherichia coli* strains carried by people may have been originated in food animals, particularly from poultry (Johnson et al., 2006).

In this context and to preserve the effectiveness of important human drugs (Casewell et al., 2003) the FDA prohibited the use of fluoroquinolones in chickens and turkeys in the United States, based on evidence that use of these antimicrobials in poultry caused development of resistance of thermofilic *Campylobacter* species. These resistant strains can be transmitted to humans with consequences to public health (Nelson et al., 2007). Selection of resistance in non-pathogenic bacteria is another potential risk. Some resistance genes may be present in non-pathogenic bacteria and then can be transferred to pathogenic microorganisms. Fairchild et al. (2005) showed that the oral administration of tetracycline did not induce significant changes in the chicken cecal bacterial community but they found that *Enterococcus* spp. susceptibility tests showed an increase on tetracycline MICs. These bacteria were positive for resistance genes, tet(M), tet(L), tet(K), and tet(O), which can be transferred to *Campylobacter jejuni*, conferring tetracycline resistance. The authors suggested that complex ecological and genetic factors could contribute to the prevalence and transfer of antibiotic resistance genes in the chicken production environment.

Despite the inconvenience of adding AGP to feed, it is generally accepted that intensification in modern poultry production and the increase in related stressors, e.g., feed changes or diet imbalances, may have different negative effects on animal health, e. g., reduced immune functions, high exposed susceptible population (Pinchasov and Noy, 1993). This may predispose broilers to colonization of the gastrointestinal tract by bacterial pathogens, producing a threat to bird’s health and food safety. Removal of antibiotic AGP from animal diets implies a tremendous pressure on the livestock and poultry farmers, one of the main consequences being a substantial increase in the incidence of infectious diseases with the associated augment in the use of antibiotics for therapy (Inborr, 2001; Casewell et al., 2003; Grave et al., 2006). *Salmonella* spp., *Campylobacter jejuni* and *Clostridium perfringens* are considered to be the most important emerging and increasing threat for poultry and human health (Van Immerseel et al., 2004; Humphrey et al., 2007). The challenge is to implement new alternatives without affecting the production performances of livestock and also avoid the increasing of antimicrobial resistance.

Alternatives to AGPs had its origin in public health programs where nutritional interventions such as probiotics and prebiotics are used to ameliorate chronic human conditions such as inflammatory bowel disease (Guarner et al., 2002; Damaskos and Kolios, 2008) and irritable bowel syndrome (Fooks and Gibson, 2002). Formulation of diets focused on specific effects on gut health is becoming a reality in the monogastric animal industries because the maintenance or enhancement of gut health is essential for the welfare and productivity of animals when antibiotics are not allowed in feed. In this scenario raw plant extracts and derived tannins are showing promising results for food animal production (Huyghebaert et al., 2011).

## PLANT EXTRACTS AND TANNINS

Plants synthesize many aromatic substances, most of which are secondary metabolites. In many cases, these substances serve as plant defense mechanisms against predation. Some, such as terpenoids, give plants their odors; others (quinones and tannins) are responsible for plant pigment, others are responsible for plant flavor (e.g., the terpenoid capsaicin from chili peppers). Tannins are water-soluble polyphenolic compounds of variable molecular weights abundantly found in nature which have the ability to precipitate proteins (Spencer et al., 1988; Cowan, 1999). Tannins can be classified into condensed and hydrolysable (Scalbert, 1991; Haslam, 1996). Hydrolyzable tannins are based on gallic acid, usually as multiple esters with D-glucose, while the more numerous condensed tannins (often called proanthocyanidins) are derived from flavonoid monomers. Current scientific evidence suggests that there is significant potential in the use of tannins to enhance nutrition and animal health, particularly for ruminants such as cattle (Frutos et al., 2004). Many studies of phenolic compounds (resveratrol, quercetin, rutin, catechin, proanthocyanidins) have been present in the last few years, most of these works were directed to improvements of human health and they demonstrate that tannins have multiple biological activities, including cardioprotective, anti-inflammatory, anti-carcinogenic, antiviral, and antibacterial properties attributed mainly to their antioxidant and antiradical activity (Frankel et al., 1993; Teissedre et al., 1996; Santos-Buelga and Scalbert, 2000). Recent studies in veterinary medicine mention that these effects are reflected in a better growth performance in different species of food producer animals. Tannins are also able to reduce the risk of livestock disease and transmission of zoonotic pathogens in a sustainable and environmentally friendly manner. Recent reports of the use of tannin in poultry show promising results (Van Parys et al., 2010; Anderson et al., 2012; Redondo et al., 2013b; Tosi et al., 2013).

## HISTORICAL CONSIDERATION OF TANNINS AS ANTI-NUTRITIONAL FACTORS

Traditional concepts in poultry nutrition consider tannins as anti-nutritional factors. In contrast with the effect on ruminant animals where tannins in the diet may have considerable nutritional benefits, tannins are generally considered undesirable in simple-stomached animals feed. In monogastric farm animals it is commonly accepted that dietary tannins reduce digestibility (in particular of crude protein) and consequently growth performance (Treviño et al., 1992; Smulikowska et al., 2001). In poultry, a considerable number of publications have shown the anti-nutritional effects of tannins in chicken feeding; these substances induce a worsening of productive performances as a consequence of decreasing voluntary feed intake and organic matter digestibility, especially the protein component (Barroga et al., 1985; Longstaff and McNab, 1991; Garcia et al., 2004; Longstaff and McNab, 2007).

Reports of anti-nutritional effects of tannins are mostly based on assays performed with relatively high concentrations of tannins in feed, mainly using purified condensed tannins or plant with excess of tannins as may be the tannic acid from sorghum grains. These experiments showed adverse effects such as decreased nutrient utilization, animal productivity, and death in certain animals. This limited experimental information and the fact that tannins act as a defense mechanism in plants against herbivores have been the origin of the widespread concept that tannins are negative for animals. However, it is now known that their beneficial or detrimental properties depend upon their chemical structure (generally associated with the plant origin) and dosage, besides other factors such as animal species, the physiological state of the animal and composition of the diet. More recent evidence suggest that a moderate tannin level is able to improve both nutrition and health status in monogastric animals.

## IMPACT ON POULTRY PRODUCTIVE PERFORMANCE

Despite that tannins have been traditionally considered as anti-nutritional factors, is it now known that these substances can be beneficial to poultry. However, as it previously mentioned, several factors must be considered and evaluated such as the final concentration in feed, the structure of the compounds, the applied process during feed preparation, and plant factors, which may affect final tannin impact on birds digestive function and global health (Hagerman and Butler, 1980). Studies with different purified tannins confirm that chemical properties, like astringent taste and protein binding are variable among tannin extracts (Hofmann et al., 2006). Schiavone et al. (2008) showed that the use of chestnut extract in poultry feeding does not influence feed digestibility, carcass quality or nitrogen balance. In fact, it has a positive influence in growth performance if included in the diet up to 0.2% (on dry matter). Similarly Marzoni et al. (2005) studied the dietary effects of quebracho tannins in growing pheasants and demonstrated that the inclusion of 2% in feed did not affect growth performances. Furthermore, some authors mention that administration of chestnut tannins may change the droppings consistency, resulting in firmer droppings in treated groups which positively affect the litter status and thus improving the overall health status and welfare of chickens in intensive production systems. Moreover, the chestnut fruit content of phenolics (gallic and ellagic acid), which have been linked to various positive effects on human health such as antioxidant activity, a decrease in the risk of cardiovascular diseases, anticancer mechanisms, and anti-inflammatory properties (de Vasconcelos et al., 2010). Tannins also can be used in combination with other AGPs alternatives, as probiotics, showing a synergist effect in the promotion of gut health. A recent work reported that chestnut extracts exhibited a surprising effect in improving the tolerance to gastric transit of Lactobacilli, while chestnut fiber mainly improved the tolerance to bile juice (Blaiotta et al., 2013).

Although tannins can have beneficial effects on the digestion and therefore animal performance when incorporated into animal diets, their primary mode of action is often not sufficiently known to explain the final *in vivo* effects. Some authors suggest that low concentration of tannins can improve palatability of feed and raise performance of monogastrics by stimulating feed intake (Windisch and Kroismayr, 2006). Others suggest that stimulation of digestive secretions is often considered to be a core mode of action (e.g. Lee et al., 2003). Nevertheless, antimicrobial properties seem to be the most relevant mode of action, especially in young animals. In general terms, like AGPs, plant derived compounds would be involved in the modulation of the highly complex interaction between microbiota and the gastrointestinal tract. The resulting relief of the animal host from microbial activity and their undesired products might be responsible for the lower immune defense costs (Windisch and Kroismayr, 2006; Kroismayr et al., 2008). However, the complexity of the interactions and dynamics of the gut microbiota makes it very difficult to define such effects in quantitative terms.

## IMPACT ON POULTRY HEALTH

Over the last few years, the dietary role of tannins is receiving increasing interest as they may reduce the number of gastrointestinal parasites in mammals (Athanasiadou et al., 2000; Butter et al., 2002; Min et al., 2005) and birds (Marzoni et al., 2005). Tannins, such as condensed tannins from green tea or quebracho, have proven to have antimicrobial activity (Sakanaka et al., 2000; Elizondo et al., 2010) and affect gastrointestinal bacteria colonization in chickens and pigs (Hara et al., 1995; Hara, 1997). Multiple reports suggest the efficacy of tannins or plant extracts in the control of zoonotic pathogens like *Campylobacter* and *Salmonella*.

*Campylobacter* spp. is one of the leading sources of human bacterial diarrhea worldwide, with *Campylobacter jejuni* and *Campylobacter coli* representing the most frequently involved species (Adak et al., 1995; Kapperud et al., 2003). One of the main sources of infection is considered to be foods of poultry origin, intestinal carriage rate within individual flocks often exceed 80% (Anderson et al., 2012). Before AGP banning in determined countries an increase in the incidence of antimicrobial resistance was observed in this food borne pathogen (Desmonts et al., 2004). The antimicrobial activity of various hydrolysable and condensed tannin-rich extracts against *Campylobacter jejuni* reveals that both types of tannins inhibit the growth of this bacterium (Nohynek et al., 2006; Gutierrez-Banuelos et al., 2011; Anderson et al., 2012). It has been observed that condensed tannins may be less efficient than hydrolysable tannins in controlling *Campylobacter jejuni* when high concentrations of amino acids and soluble proteins are present (Anderson et al., 2012). The efficacy of adding selected tannins to poultry feed to diminish the *in vivo* incidence of *Campylobacter* spp., needs to be evaluated.

*Salmonella* serovar Enteritidis is one of the foodborne pathogens most commonly associated with the consumption of poultry products. Control strategies of the disease in humans are based on reducing contamination during slaughter and *Salmonella* *Enteritidis* load in birds. This was achieved with the use of AGP, which generates residues in meat and eggs and favors the selection of multi-resistant strains of *Salmonella* and other pathogens. Van Parys et al. (2010) found that chestnut (*Castanea sativa*) derived tannins were able to inhibit the *in vitro *growth of *Salmonella typhimurium*, but had no effect on the excretion of the bacteria in an infection model in pigs. Quebracho (*Schinopsis lorentzii*) raw extract shows bacteriostatic effect on *Salmonella Enteritidis* *in vitro*, and when used in an experimental infection model in broilers it was able to reduce the excretion of the bacteria (Redondo et al., 2013a). Similarly, Prosdócimo et al. (2010) found antibacterial activity of quebracho against *Salmonella* *Enteritidis* and *Salmonella* Gallinarum *in vitro*.

*Clostridium perfringens* is considered an important poultry pathogen that is the causative agent of necrotic enteritis and sub-clinical disease (Ficken and Wages, 1997). Both presentations of the disease have important economic impact on poultry production. This bacterium is an important example of antimicrobial banning consequences. AGPs have long been effective in prevention of necrotic enteritis in poultry flocks and after AGPs withdrawal, the incidence of necrotic enteritis increased considerably (Van Immerseel et al., 2004). Inhibitory effects of tannins from different sources have been demonstrated. Previous report shows that tannins derived from chestnut and quebracho have *in vitro* antibacterial and antitoxin activities against *Clostridium perfringens* and its toxins and that mixtures of both tannins maintain individual activities (Elizondo et al., 2010). Subsequent results from this research group confirm the *in vivo* effects of chestnut and quebracho tannins in a broiler necrotic enteritis model reducing the incidence and severity of gross lesions and improving the productive performance of broiler chickens (Redondo et al., 2013b). This findings are reinforced by the results obtained from other authors with chestnut tannin added to diet in an *Eimeria *spp./*Clostridium perfringens* co-infection model (Tosi et al., 2013). Although chestnut tannins show strong bactericidal activity against *Clostridium perfringens*, most ingested tannin do not remain in the feces because it is hydrolyzed and degraded in the intestinal tract. In contrast, quebracho tannins are mainly condensed with lower antibacterial ability but most of the administered tannins remain in the fecal material. Therefore, those different abilities could be used to readily diminish the intestinal *Clostridium perfringens* load by chestnut and avoiding the reinfection by controlling the environmental contamination (i.e. feces and bedding) with quebracho tannins.

Different works reports the antiviral activity of some tannin against animal viruses. Ueda et al. (2013) test condensed and hydrolysable tannins from different sources against selected families of pathogenic animal virus and show that these compounds have an unspecific neutralizing effect on enveloped virus. The same group reports the induced aggregation of purified virions or BSA through association of tannins with proteins. Another potential mechanism was reported in works using human virus, like herpesvirus (Lin et al., 2011) and human immunodeficiency virus (HIV-1), in the same the authors suggest that reduce viral activity could be due to tannins binding to cell receptor like glycoproteins or CD4, respectively. Although they are few, works with avian viruses suggest that natural extracts containing specific tannins could contribute to control viral infections. Lupini et al. (2009) showed that both, chestnut and quebracho wood extracts, have inhibitory effect on avian reovirus (retrovirus) and avian metapneumovirus (paramyxovirus) before virus absorption to cells. In this work the author reports that chestnut and quebracho extracts reduce the extracellular viral activity, proposing that extracellular effect may be due to an interaction between tannins and viral proteins resulting in the inhibition of viral attachment and penetration of the cell membrane, as mention before for other virus. In the same work, they report a reduction in the intracellular viral activity only by quebracho extract, and propose that the main mechanism would be the inhibition of viral enzymes. The higher intracellular activity of quebracho extract could be due to the smaller size of tannins extracted from this plant that could penetrate the cells as suggested by Moreira et al. (2005).

Although tannins or plant derived extracts demonstrated activity against viral (Lupini et al., 2009), bacterial (Tosi et al., 2013), and protozoal diseases (Cejas et al., 2011), little is known about the mechanisms of these compounds on antimicrobial effects and growth promotion. Some of the explained modes of action for antimicrobials may help to define tannins main mechanism. Metabolism inhibition is one possible mechanism; Bae et al. (1993) showed that condensed tannins from birdfoot trefoil (*Lotus corniculatus* L.) were inhibitory to the endoglucanase activity of cellulose digesting *Fibrobacter succinogenes* S85 in the rumen. This may be applied to virulence factors as Elizondo suggests for *Clostridium perfringens* toxins (2010). On the other hand, iron deprivation has been suggested by some authors (Scalbert, 1991; Haslam, 1996; Mila et al., 1996). Tannic acid works like a siderophore to chelate iron from the medium, making it unavailable for the microorganisms. Iron is essential for most pathogenic bacteria and tannic acid shows three times more affinity for iron than *E. coli* siderophores (Chung et al., 1998).

One of the most accepted mechanisms of action of some plant tissues in animal diet is the shifts in intestinal microbiota composition. As reported for different groups, Gram positive bacteria seem to be more sensitive to plant extract with high tannins content (Nohynek et al., 2006; Engels et al., 2011). It is important to remark that microbiota changes have more impact on younger animals due to their constantly evolving microbiota. It is thought to take until the sixth week of age to achieve a mature microbiota (Barnes et al., 1972). Regardless of the mode of action, the chemical characteristics of the tannins are highly variable and different types of tannins can be present in one plant extract. Therefore, the origin of the plant extract added to the feed will be determinant in the final impact on microbiota and the animal performance.

## ECONOMICAL CONSIDERATION

Independently of the use of antimicrobials or any of the available alternatives to AGP, increase of the productive performance and animal welfare depends on the overall health status of broilers. A complete and continuous observation of the flock health status and performance must be considered. It requires regular necropsies, sampling, and identification of pathogens, together with periodic monitoring of productive parameters like feed intake and weight gain, flock uniformity and other conditions. This should provide an overview of the productive costs and allow measure the economic impact of a disease and choose cost-effective therapeutic or prophylactic strategies. The return-on-investment for alternatives to AGPs will depend on both the biological impact and the dynamics of the market price. Withdraw of AGP from the flock may cause a decreased growth rate, higher morbidity, and mortality; but the continuous use may led to increased condemnations due to residues in meat and derived products. In countries where the use of AGP is still allowed is imperative to consider the net economic effect of replacing them with alternative products that do not represent a threat to public health and leave no residues in meat and derived products. It will depend on several factors including impact effects on productive performance levels and the cost of any alternative potential technologies adopted to compensate for the termination of use of AGPs and may be offset by the benefits like access to more demanding markets or differential marketing, as in the case of organic foods. In countries where AGP are banned from poultry production, the negative impact may be temporarily compensated by the use of ionophore anticoccidials, which are excluded from regulation due to lack of reports of relations between these substances and others antimicrobials. Taking into consideration that recent field investigations have demonstrated that animal husbandry use of antimicrobial agents increases the likelihood that domestic animal bacteria will develop resistance or cross-resistance to drugs approved for use in human medicine (Diarra et al., 2007; da Costa et al., 2013), experience with others groups of antimicrobials suggest that these chemicals are prompt to be removed from animal feed for the same reasons as AGPs, it is important to develop adequate alternatives to be used alone or combined with other control measures to improve the gut health. Further work is needed to define standards for the replacement of antibiotic compounds in poultry in terms of product type, identification of suppliers, poultry response criteria, regulatory status and veterinary definition (Rosen, 2003).

## CONCLUSION

The use of plant extracts appears as an attractive alternative to the use of antimicrobial growth promoter factors. These natural products do not leave residues in poultry-derived products. Also, plant extracts are complex substances with many bioactive principles that would have fewer chances to induce resistance in microorganisms.

Successful application of any of these extracts as feed additive must ensure a product of consistent quality in enough quantities to improve poultry production at AGPs levels and fulfill the actual requirements of poultry derived products consumers. If the products are effective and can be acquired in enough quantities to supply the poultry industry requirements, the decisive factor for the successful application will be the cost and it should be at least similar to those of the AGPs. Although numerous products available in market have been proved to be efficient in the field (Graziani et al., 2006; Lupini et al., 2009; Elizondo et al., 2010; Redondo et al., 2013b), many have less clear potential. Chestnut (hydrolizable) and Quebracho (condennsed) tannins are probably the most readily available commercial products that are being used and cover those needs as well as there is an important number of data supporting their usage.

The diversity of results presented in different papers show the complexity of elucidating effects of plant extracts over a determined microorganism or disease in different animal hosts. Further investigations needs to be done in order to describe the effects of plant extracts on pathogenic microorganism as well as in commensal microbiota and the impact of its use in animal production. This knowledge would allow the development of new and innovative products suitable to be incorporated in animal feed in order to improve animal production without compromising public health.

## Conflict of Interest Statement

The authors declare that the research was conducted in the absence of any commercial or financial relationships that could be construed as a potential conflict of interest.

## References

[B1] Adak G. K. Cowden J. M. Nicholas S. (1995). The Public Health Laboratory Service national case-control study of primary indigenous sporadic cases of *Campylobacter* infection. *Epidemiol. Infect.* 115 15–22 10.1017/S0950268800058076 7641828 PMC2271554

[B2] Anderson R. C. Vodovnik M. Min B. R. Pinchak W. E. Krueger N. A. Harvey R. B. (2012). Bactericidal effect of hydrolysable and condensed tannin extracts on *Campylobacter jejuni* in vitro. *Folia Microbiol.* 57 253–258 10.1007/s12223-012-0119-4 22528299

[B3] Athanasiadou S. Kyriazakis I. Jackson F. Coop R. L. (2000). Effects of short-term exposure to condensed tannins on adult *Trichostrongylus colubriformis*. *Veter. Record* 146 728–732 10.1136/vr.146.25.728 10901215

[B4] Bae H. D. McAllister T. A. Yanke J. Cheng K.-J. Muir A. D. (1993). Effects of condensed tannins on endoglucanase activity and filter paper digestion by *Fibrobacter succinogenes* S85. *Appl. Environ. Microbiol.* 59 2132–2138 16348990 10.1128/aem.59.7.2132-2138.1993 PMC182247

[B5] Barnes E. M. Mead G. C. Barnum D. A. Harry E. G. (1972). The intestinal flora of the chicken in the period 2 to 6 weeks of age, with particular reference to the anaerobic bacteria. *Br. Poult. Sci.* 13 311–326 10.1080/00071667208415953 4555258

[B6] Barroga C. F. Laurena A. C Mendoza E. M. T. (1985). Effect of condensed tannins on the in vitro protein digestibility of mungbean (*Vigna radiata* (L.)* Wilczek*). *J. Agric. Food Chem.* 33 1157–1159 10.1021/jf00066a033

[B7] Blaiotta G. La Gatta B. Di Capua M. Di Luccia A. Coppola R. Aponte M. (2013). Effect of chestnut extract and chestnut fiber on viability of potential probiotic *Lactobacillus* strains under gastrointestinal tract conditions. *Food Microbiol.* 36 161–169 10.1016/j.fm.2013.05.002 24010594

[B8] Butaye P. Devriese L. A. Haesebrouck F. (2003). Antimicrobial growth promoters used in animal feed: effects of less well known antibiotics on gram-positive bacteria. *Clin. Microbiol. Rev.* 16 175–188 10.1128/CMR.16.2.175-188.2003 12692092 PMC153145

[B9] Butter N. L. Dawson J. M. Wakelin D. Butter P. J. (2002). Effect of dietary condensed tannins on gastrointestinal nematodes. *J. Agric. Sci.* 137 461–469

[B10] Casewell M. Friis C. Marco E. McMullin P. Phillips I. (2003). The European ban on growth-promoting antibiotics and emerging consequences for human and animal health. *J. Antimicrob. Chemother.* 52 159–161 10.1093/jac/dkg313 12837737

[B11] Cebra J. J. (1999). Influences of microbiota on intestinal immune system development. *Am. J. Clin. Nutr.* 69 1046S–1051S 10232647 10.1093/ajcn/69.5.1046s

[B12] Cejas E. Pinto S. Prosdocimo F. Batalle M. Barrios H. Tellez G. M. (2011). Evaluation of quebracho red wood (*Schinopsis lorentzii*) polyphenolic vegetable extracts for the reduction of coccidiosis in broiler chicks. *Int. J. Poultry Sci.* 10 344–349 10.3923/ijps.2011.344.349

[B13] Chung K.-T. Lu Z. Chou M. (1998). Mechanism of inhibition of tannic acid and related compounds on the growth of intestinal bacteria. *Food Chem. Toxicol.* 36 1053–1060 10.1016/S0278-6915(98)00086-6 9862646

[B14] Collignon P. Angulo F. J. (2006). Fluoroquinolone-resistant *Escherichia coli*: food for thought. *J. Infect. Dis.* 194 8–10 10.1086/504922 16741876

[B15] Cowan M. (1999). Plant products as antimicrobial agents. *Clin. Microbiol. Rev.* 12 564–582 10515903 10.1128/cmr.12.4.564 PMC88925

[B16] da Costa P. M. Loureiro L Matos A. J. F. (2013). Transfer of multidrug-resistant bacteria between intermingled ecological niches: the interface between humans, animals and the environment. *Int. J. Environ. Res. Public Health* 10 278–294 10.3390/ijerph10010278 23343983 PMC3564142

[B17] Damaskos D. Kolios G. (2008). Probiotics and prebiotics in inflammatory bowel disease: microflora “on the scope”. *Br. J. Clin. Pharmacol.* 65 453–467 10.1111/j.1365-2125.2008.03096.x 18279467 PMC2291386

[B18] Desmonts M.-H. Dufour-Gesbert F. Avrain L. Kempf I. (2004). Antimicrobial resistance in *Campylobacter* strains isolated from French broilers before and after antimicrobial growth promoter bans. *J. Antimicrob. Chemother.* 54 1025–1030 10.1093/jac/dkh473 15537699

[B19] de Vasconcelos M. do C. B. M. Bennett R. N. Quideau S. Jacquet R. Rosa E. A. S. (2010). Evaluating the potential of chestnut (*Castanea sativa* Mill.) fruit pericarp and integument as a source of tocopherols, pigments and polyphenols. *Ind. Crops Prod.* 31 301–311 10.1016/j.indcrop.2009.11.008

[B20] Devirgiliis C. Zinno P. Perozzi G. (2013). Update on antibiotic resistance in foodborne *Lactobacillus* and *Lactococcus* species. *Front. Microbiol.* 4:301 10.3389/fmicb.2013.00301 PMC3792357 24115946

[B21] Diarra M. S. Silversides F. G. Diarrassouba F. Pritchard J. Masson L. Brousseau R. (2007). Impact of feed supplementation with antimicrobial agents on growth performance of broiler chickens, *Clostridium perfringens* and *Enterococcus *counts, and antibiotic resistance phenotypes and distribution of antimicrobial resistance determinants in Escheric. *Appl. Environ. Microbiol.* 73 6566–6576 10.1128/AEM.01086-07 17827305 PMC2075079

[B22] Dibner J. J. Richards J. D. (2005). Antibiotic growth promoters in agriculture: history and mode of action. *Poult. Sci.* 84 634–643 10.1093/ps/84.4.634 15844822

[B23] Elizondo A. M. Mercado E. C. Rabinovitz B. C Fernandez Miyakawa M. E. (2010). Effect of tannins on the in vitro growth of *Clostridium perfringens*. *Veter. Microbiol.* 145 308–314 10.1016/j.vetmic.2010.04.003 20471759

[B24] Engels C. Schieber A Gänzle M. G. (2011). Inhibitory spectra and modes of antimicrobial action of gallotannins from mango kernels (*Mangifera indica* L.). *Appl. Environ. Microbiol.* 77 2215–2223 10.1128/AEM.02521-10 21317249 PMC3067452

[B25] Fairchild A. S. Smith J. L. Idris U. Lu J. Sanchez S. Purvis L. B. (2005). Effects of orally administered tetracycline on the intestinal community structure of chickens and on tet determinant carriage by commensal bacteria and *Campylobacter jejuni*. *Appl. Environ. Microbiol.* 71 5865–5872 10.1128/AEM.71.10.5865-5872.2005 16204498 PMC1265988

[B26] Ficken M. D. Wages D. P. (1997). “Necrotic enteritis,” in *Diseases of Poultry* 10th Edn ed. Calnek B.W. (Ames, IA:Mosby-Wolfe) 261–264

[B27] Fooks L. J. Gibson G. R. (2002). Probiotics as modulators of the gut flora. *Br. J. Nutr. *88(Suppl. 1) S39–S49 10.1079/BJN2002628 12215180

[B28] Frankel E. German J. Kinsella J. Parks E. Kanner J. (1993). Inhibition of oxidation of human low-density lipoprotein by phenolic substances in red wine. *Lancet* 341 454–457 10.1016/0140-6736(93)90206-V 8094487

[B29] Frutos P. Hervás G. Giráldez F. J Mantecón, A. R. (2004). Review. Tannins and ruminant nutrition Tannins?: Structure and chemical. *Span. J. Agric. Res.* 2 191–202

[B30] Garcia R. Mendes A. Sartori J. Paz I. Takahashi S. Pelícia K. (2004). Digestibility of feeds containing sorghum, with and without tannin, for broiler chickens submitted to three room temperatures. *Braz. J. Poultry Sci.* 6 55–60 10.1590/S1516-635X2004000100007

[B31] Gaskins H. R. Collier C. T. Anderson D. B. (2002). Antibiotics as growth promotants: mode of action. *Anim. Biotechnol.* 13 29–42 10.1081/ABIO-120005768 12212942

[B32] Grave K. Jensen V. F. Odensvik K. Wierup M. Bangen M. (2006). Usage of veterinary therapeutic antimicrobials in Denmark, Norway and Sweden following termination of antimicrobial growth promoter use. *Prev. Vet. Med.* 75 123–132 10.1016/j.prevetmed.2006.02.003 16580756

[B33] Graziani R. Tosi G. Denti R. (2006). In vitro antimicrobial activity of SILVA FEED ENC on bacterial strains of poultry origin. *In EPC 2006 – 12th European Poultry Conference*. World’s Poultry Science Association Verona

[B34] Guarner F. Casellas F. Borruel N. Antolín M. Videla S. Vilaseca J. (2002). Role of microecology in chronic inflammatory bowel diseases. *Eur. J. Clin. Nutr. *56(Suppl. 4) S34–S38 10.1038/sj.ejcn.1601662 12556945

[B35] Gutierrez-Banuelos H. Pinchak W. E. Min B. R. Carstens G. E. Anderson R. C. Tedeschi L. O. (2011). Effects of feed-supplementation and hide-spray application of two sources of tannins on enteric and hide bacteria of feedlot cattle. *J. Environ. Sci. Health B Pest. Food Contamin. Agric. Wastes* 46 360–365 10.1080/03601234.2011.559419 21547824

[B36] Hagerman A. E. Butler L. G. (1980). Determination of protein in tannin-protein precipitates. *J. Agric. Food Chem.* 28 944–947 10.1021/jf60231a010 7462521

[B37] Hara Y. (1997). Influence of tea catechins on the digestive tract. *J. Cell. Biochem.* 67 52–58 10.1002/(SICI)1097-4644(1997)27+<52::AID-JCB10>3.0.CO;2-N 9591193

[B38] Hara H. Orita N. Hatano S. Ichikawa H. Hara Y. Matsumoto N. (1995). Effect of tea polyphenols on fecal flora and fecal metabolic products of pigs. *J. Veter. Med. Sci.* 57 45–49 10.1292/jvms.57.45 7756423

[B39] Haslam E. (1996). Natural polyphenols (vegetable tannins) as drugs: possible modes of action. *J. Nat. Prod.* 59 205–215 10.1021/np960040+ 8991956

[B40] Hofmann T. Glabasnia A. Schwarz B. Wisman K. N. Gangwer K. A. Hagerman A. E. (2006). Protein binding and astringent taste of a polymeric procyanidin, 1,2,3,4,6-penta-O-galloyl-beta-D-glucopyranose, castalagin, and grandinin. *J. Agric. Food Chem.* 54 9503–9509 10.1021/jf062272c 17147439 PMC2597504

[B41] Humphrey T. O’Brien S. Madsen M. (2007). *Campylobacters* as zoonotic pathogens: a food production perspective. *Int. J. Food Microbiol.* 117 237–257 10.1016/j.ijfoodmicro.2007.01.006 17368847

[B42] Huyghebaert G. Ducatelle R Van Immerseel F. (2011). An update on alternatives to antimicrobial growth promoters for broilers. *Veter. J.* 187 182–188 10.1016/j.tvjl.2010.03.003 20382054

[B43] Inborr J. (2001). Sweedish poultry production witgout in-feed antibiotics. A oportunity to clostridia.* Br. Poultry Sci.* 42 64–68

[B44] Johnson J. R. Kuskowski M. A. Menard M. Gajewski A. Xercavins M. Garau J. (2006). Similarity between human and chicken *Escherichia coli* isolates in relation to ciprofloxacin resistance status. *J. Infect. Dis.* 194 71–78 10.1086/504921 16741884

[B45] Jukes T. H. Stokstad E. L. R. Taylor R. R. Cunha T. J. Edwards H. M. Meadows G. B. (1950). Growth-promoting effect of aureomycin on pigs. *Arch. Biochem.* 26 324–325 15411210

[B46] Kapperud M. Espeland G. Wahl E. Walde A. Herikstad H. Gustavsen S. (2003). Factors associated with increased and decreased risk of *Campylobacter* infection: a prospective case-control study in Norway. *Am. J. Epidemiol.* 158 234–242 10.1093/aje/kwg139 12882945

[B47] Kazimierczak K. A. Flint H. J. Scott K. P. (2006). Comparative analysis of sequences flanking tet(W) resistance genes in multiple species of gut bacteria. *Antimicrob. Agents Chemother.* 50 2632–2639 10.1128/AAC.01587-05 16870752 PMC1538676

[B48] Kelly D. Conway S. (2005). Bacterial modulation of mucosal innate immunity. *Mol. Immunol.* 42 895–901 10.1016/j.molimm.2004.12.003 15829279

[B49] Kroismayr A. Sehm J. Pfaffl M. W. Schedle K. Plitzner C Windisch W. (2008). Effects of avilamycin and essential oils on mRNA expression of apoptotic and inflammatory markers and gut morphology of piglets. *Czech J. Veter.* 9 377–387

[B50] Landers T. F. Cohen B. Wittum T. E. Larson E. L. (2012). A review of antibiotic use in food animals: perspective, policy, and potential. *Publ. Health Rep.* 127 4–22 10.1177/003335491212700103 PMC3234384 22298919

[B51] Lee K. W. Everts H. Kappert H. J. Frehner M. Losa R. Beynen A. C. (2003). Effects of dietary essential oil components on growth performance, digestive enzymes and lipid metabolism in female broiler chickens. *Br. Poult. Sci.* 44 450–457 10.1080/0007166031000085508 12964629

[B52] Lin L.-T. Chen T.-Y. Chung C.-Y. Noyce R. S. Grindley T. B. McCormick C. (2011). Hydrolyzable tannins (chebulagic acid and punicalagin) target viral glycoprotein-glycosaminoglycan interactions to inhibit herpes simplex virus 1 entry and cell-to-cell spread. *J. Virol.* 85 4386–4398 10.1128/JVI.01492-10 21307190 PMC3126266

[B53] Longstaff M. McNab J. M. (1991). The inhibitory effects of hull polysaccharides and tannins of field beans (*Vicia faba* L.) on the digestion of amino acids, starch and lipid and on digestive enzyme activities in young chicks. *Br. J. Nutrit.* 65 199–216 10.1079/BJN19910081 1645991

[B54] Longstaff M. A. McNab J. M. (2007). The effect of concentration of tannin-rich bean hulls (*Vicia faba* L.) on activities of lipase (EC 3.1.1.3) and α-amylase (EC 3.2.1.1) in digesta and pancreas and on the digestion of lipid and starch by young chicks. *Br. J. Nutrit.* 66 139 10.1079/BJN19910017 1931901

[B55] Lupini C. Cecchinato M. Scagliarini A. Graziani R. Catelli E. (2009). In vitro antiviral activity of chestnut and quebracho woods extracts against avian reovirus and metapneumovirus. *Res. Veter. Sci.* 87 482–487 10.1016/j.rvsc.2009.04.007 19435637

[B56] Marzoni M. Castillo A. Romboli I. (2005). Effect of dietary inclusion of quebracho (*Schinopsis lorentzii*) tannins on productive performances of growing pheasant females. *Ital. J. Anim. Sci.* 4(Suppl. 2) 507–509

[B57] Mathur S. Singh R. (2005). Antibiotic resistance in food lactic acid bacteria – a review. *Int. J. Food Microbiol.* 105 281–295 10.1016/j.ijfoodmicro.2005.03.008 16289406

[B58] Mila I. Scalbert A. Expert D. (1996). Iron withholding by plant polyphenols and resistance to pathogens and rots. *Phytochemistry* 42 1551–1555 10.1016/0031-9422(96)00174-4

[B59] Min B. R. Hart S. P. Miller D. Tomita G. M. Loetz E. Sahlu T. (2005). The effect of grazing forage containing condensed tannins on gastro-intestinal parasite infection and milk composition in Angora does. *Veter. Parasitol.* 130 105–113 10.1016/j.vetpar.2005.03.011 15893077

[B60] Moore P. R. Evenson A. (1946). Use of sulfasuxidine, streptothricin, and streptomycin in nutritional studies with the chick. *J. Biol. Chem.* 165 437–441 20276107

[B61] Moreira M. R. Ponce A. G. de Valle C. E. Roura S. I. (2005). Inhibitory parameters of essential oils to reduce a foodborne pathogen. *Lebensmittel Wissenschaft Technol.* 38 565–570 10.1016/j.lwt.2004.07.012

[B62] Nelson J. M. Chiller T. M. Powers J. H. Angulo F. J. (2007). Fluoroquinolone-resistant *Campylobacter* species and the withdrawal of fluoroquinolones from use in poultry: a public health success story. *Clin. Infect. Dis.* 44 977–980 10.1086/512369 17342653

[B63] Nohynek L. J. Alakomi H.-L. Kähkönen M. P. Heinonen M. Helander I. M. Oksman-Caldentey K.-M. (2006). Berry phenolics: antimicrobial properties and mechanisms of action against severe human pathogens. *Nutr. Cancer* 54 18–32 10.1207/s15327914nc5401_4 16800770

[B64] Page S. W. (2006). “Current use of antimicrobial growth promoters in food animals: The benefits,” in *Antimicrobial Growth Promoters: Where Do We Go from Here? Vol. 136* eds Barug D. de Jong J. Kies A. K. Verstegen M. (Wageningen: Wageningen Academic Publishers) 19–51

[B65] Pinchasov Y. Noy Y. (1993). Comparison of post – hatch holding time and subsequent early performance of broiler chicks and Turkey poults. *Br. Poult. Sci.* 34 111–120 10.1080/00071669308417567

[B66] Prosdócimo F. Batallé M. Sosa N. De Franceschi M. Barrios H. (2010). Determinación in vitro del efecto antibacteriano de un extracto obtenido de quebracho colorado, *Schinopsis lorentzii*. *InVet* 12 139–143

[B67] Redondo L. M. Fernandez Miyakawa M. E. Fortunato R. Salvat A. Chacana P. (2013a). Eficacia de aditivos alimentarios basados en extractos vegetales para disminuir la excreción de *Salmonella Enteritidis* en pollitos BB. *II Seminario Internacional de Salmonelosis aviar*. Medellin, Colombia

[B68] Redondo L. M. Redondo E. A. Delgado F. La Sala L. Pereyra A. Garbaccio S. (2013b). “Control of *Clostridium perfringens* necrotic enteritis by tannins added to the diet,” in *Proceedings of the 8th International Conference on the Molecular Biology and Pathogenesis of the Clostridia (ClostPath 8)* Vol. 5. Palm Cove

[B69] Rosen G. D. (2003). Pronutrient antibiotic replacement standards discussed. *Feedstuffs* 75 11–13

[B70] Sakanaka S. Juneja L. R. Taniguchi M. (2000). Antimicrobial effects of green tea polyphenols on thermophilic spore-forming bacteria. *J. Biosci. Bioeng.* 90 81–85 16232822 10.1016/s1389-1723(00)80038-9

[B71] Salyers A. A. Gupta A. Wang Y. (2004). Human intestinal bacteria as reservoirs for antibiotic resistance genes. *Trends Microbiol.* 12 412–416 10.1016/j.tim.2004.07.004 15337162

[B72] Santos-Buelga C. Scalbert A. (2000). Proanthocyanidins and tannin-like compounds – nature, occurrence, dietary intake and effects on nutrition and health. *J. Sci. Food Agric.* 80 1094–1117 10.1002/(SICI)1097-0010(20000515)80:7<1094::AID-JSFA569>3.0.CO;2-1

[B73] Scalbert A. (1991). Antimicrobial properties of tannins. *Phytochemistry* 30 3875–3883 10.1016/0031-9422(91)83426-L

[B74] Schiavone A. Guo K. Tassone S. Gasco L. Hernandez E. Denti R. (2008). Effects of a natural extract of chestnut wood on digestibility, performance traits, and nitrogen balance of broiler chicks. *Poult. Sci.* 87 521–527 10.3382/ps.2007-00113 18281579

[B75] Smulikowska S. Pastuszewska B. Swiech E. Ochtabinska A. Mieczkowska A. Nguyen V. C. (2001). Tannin content affects negatively nutritive value of pea for monogastrics. *J. Anim. Feed Sci.* 10 511–523

[B76] Spencer C. M. Cai Y. Martin R. Gaffney S. H. Goulding P. N. Magnolato D. (1988). Polyphenol complexation – some thoughts and observations. *Phytochemistry* 27 2397–2409 10.1016/0031-9422(88)87004-3

[B77] Teissedre P. L. Frankel E. N. Waterhouse A. L. Peleg H. German J. B. (1996). Inhibition of in vitro human LDL oxidation by phenolic antioxidants from grapes and wines. *J. Sci. Food Agric.* 70 55–61 10.1002/(SICI)1097-0010(199601)70:1<55::AID-JSFA471>3.0.CO;2-X

[B78] Tosi G. Massi P. Antongiovanni M. Buccioni A. Minieri S. Marenchino L. (2013). Efficacy test of a hydrolysable tannin extract against necrotic enteritis in challenged broiler chickens. *Ital. J. Anim. Sci.* 12 123–132 10.4081/ijas.2013.e62

[B79] Treviño J. Ortiz L. Centeno C. (1992). Effect of tannins from faba beans (*Vicia faba*) on the digestion of starch by growing chicks. *Anim. Feed Sci. Technol.* 37 345–349 10.1016/0377-8401(92)90017-Z

[B80] Tuohy K. Rouzaud G. Bruck W. Gibson G. (2005). Modulation of the human gut microflora towards improved health using prebiotics – assessment of efficacy. *Curr. Pharm. Des.* 11 75–90 10.2174/1381612053382331 15638753

[B81] Ueda K. Kawabata R. Irie T. Nakai Y. Tohya Y. Sakaguchi T. (2013). Inactivation of pathogenic viruses by plant-derived tannins: strong effects of extracts from persimmon (Diospyros kaki) on a broad range of viruses. *PLoS ONE * 8:e55343 10.1371/journal.pone.0055343 PMC3555825 23372851

[B82] Van Immerseel F. De Buck J. Pasmans F. Huyghebaert G. Haesebrouck F. Ducatelle R. (2004). *Clostridium perfringens* in poultry: an emerging threat for animal and public health. *Avian Pathol.* 33 537–549 10.1080/03079450400013162 15763720

[B83] Van Parys A. Boyen F. Dewulf J. Haesebrouck F. Pasmans F. (2010). The use of tannins to control *Salmonella typhimurium* infections in pigs. *Zoon. Publ. Health* 57 423–428 10.1111/j.1863-2378.2009.01242.x 19538452

[B84] Wegener H. C. (2003). Antibiotics in animal feed and their role in resistance development. *Curr. Opin. Microbiol.* 6 439–445 10.1016/j.mib.2003.09.009 14572534

[B85] Windisch W. Kroismayr A. (2006). *The Effects of Phytobiotics on Performance and Gut Function in Monogastrics.* World Nutrition Forum: The Future of Animal Nutrition Vienna 85–90

